# Switching behavior of droplets crossing nodes on a fiber network

**DOI:** 10.1038/s41598-017-13009-8

**Published:** 2017-10-17

**Authors:** F. Weyer, A. Duchesne, N. Vandewalle

**Affiliations:** GRASP, Physics Department, University of Liège, Liège, Belgium

## Abstract

Lately, curious structures have been erected in arid regions: they are large nets able to catch water from fog. Tiny droplets condense on the mesh and are collected on the bottom of it. This innovative technology is crucial to obtain drinkable water in these inhospitable areas. Many studies aim to understand the behavior of droplets trapped on this entanglement of fibers. However, the motion of a droplet sliding on a network of inclined fibers and encountering several crossings when going down remains an open question. Here, we look at the path chosen by such a drop and, especially, we analyze its behavior at the different nodes of the array. We show that droplets may change from one fiber to another one depending on the slope and the diameter of these fibers. We prove that we can force a droplet to follow a specific path simply by carefully designing the fiber mesh. These findings are expected to provide a very convenient way to manipulate small droplets in applications from microfluidics to fog harvesting.

## Introduction

In nature, many organisms have or produce particular structures which turn out to be very efficient at collecting water. In arid regions, some mosses are able to collect water thanks to tiny hairs on their leaves^1^. Certain cacti can catch water droplets from fog and carry them to their stems^2^. Spiderwebs have a remarkable ability to collect water in air due to the structure of the silk^3^. All these processes involve water droplets on fiber-like structures and are widely studied. For example, Hou *et al*.^4^, Wang *et al*.^5^ and Chen *et al*.^6,7^ look at water harvesting on bio-inspired fibers.

These examples inspired new technologies like fog harvesting systems. Indeed, the collection of water in desert areas is a major challenge. An innovative way to obtain water is to collect fog droplets thanks to a large mesh net. Therefore, looking at the collection process of water droplets on inclined fibers^8^, on porous media^9^ or on fiber networks^10^ is of considerable interest. Both the design^11^ and the surface properties^12^ of the fiber mesh have been studied to improve the efficiency of the water collection. Bearing in mind these issues, it seems logical to strive for a better understanding of the behavior of droplets on fiber arrays.

Many researches focus on drops on horizontal fibers^13,14^ or on the motion of the droplets along an inclined wire^15–17^. They aim to understand how droplets deform on the fiber, how they slide, how the tilted angle influences the motion,… Gilet *et al*.^18^ study a droplet gliding along a vertical fiber and encountering several horizontal fibers. For this very specific case, they establish a criterion for determining whether the droplet crosses the node or stops. These results are used to create complex droplets on fiber networks^19^. Sauret *et al*.^20,21^ examine the geometry of silicone oil drops trapped at the meeting of two randomly oriented horizontal fibers from a static point of view. Other studies look after droplets on flexible fibers^22,23^. Even though the motion of a droplet on a vertical fiber has been addressed and the fiber inclination has been considered from a static point of view, the dynamics of a droplet on an array of randomly inclined fibers remains an open question.

**Figure 1:**
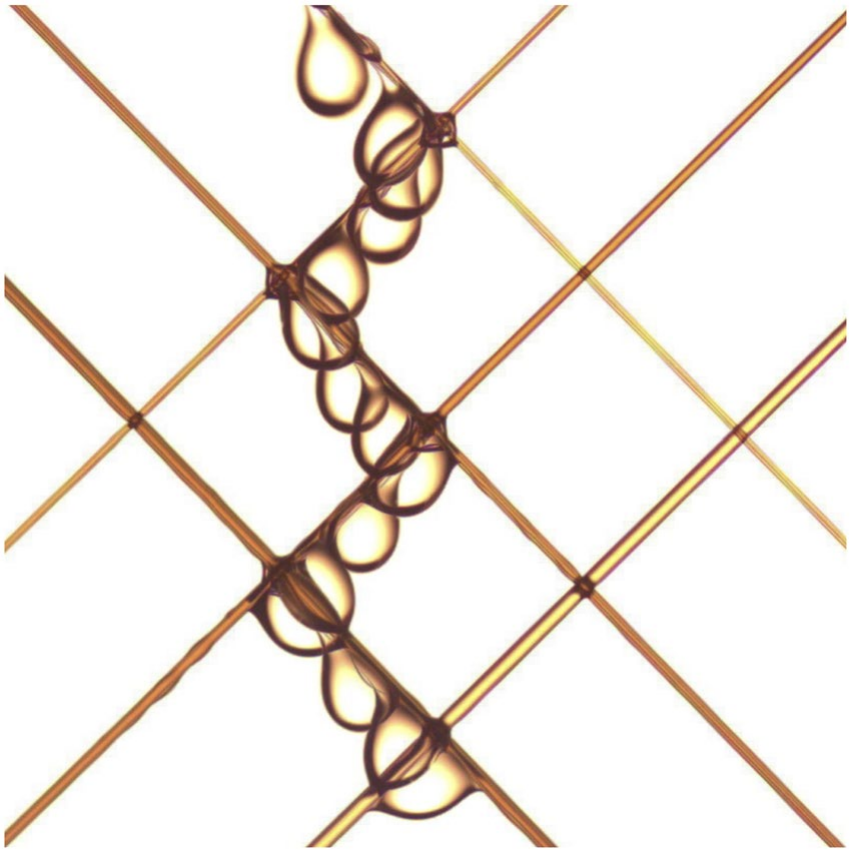
An array made of various fibers inclined at 45°: going from the top left to the bottom right, three fibers are stretched with respective diameters of 160 *μ*m, 250 *μ*m and 350 *μ*m and going from the top right to the bottom left, three other fibers are fixed with diameters of 200 *μ*m, 300 *μ*m and 450 *μ*m. A 9 *μl* droplet starts its motion from the thinnest fiber and switches from one fiber to the other. At each crossing, the oil droplet goes from one fiber to a thicker one. The droplet zigzags on the fiber network.

In this paper, we aim to understand how a droplet moves on a network of perpendicular fibers with various orientations. Especially, we look at the behavior of the droplet at the crossing of two wires. When a droplet reaches a node, it faces three different possibilities: this droplet can either be trapped at the node or go through it and if it crosses the node, the droplet can either stay on the initial fiber or change to the other one. We determine the conditions under which the droplet crosses the node and the criteria for choosing one fiber or the other after the crossing. A critical volume is found for the trapping of the droplet and seems to be related to the orientation of the crossing and the fiber diameters. Moreover, for a crossing of identical fibers, we show that the droplet detaches along the most inclined fiber. This means that it can either stay on the initial fiber or change to the other depending on their slope. A more surprising result is obtained for crossings made of two different fibers. Indeed, by changing the fiber diameters, we can force the droplet to choose the largest fiber even though it is not the steepest one. This finding involves that it is possible to force a droplet to follow a determinist path on a fiber array just by choosing the suitable set of fiber diameters. This can be seen from Fig. 1 where an oil droplet is changing from one fiber to a thicker one at each node. This particular behavior will be explain throughout this paper. We also propose to generalize our results for other fluids and other angles between the fibers of the network.

## Material and Methods

We work with a Dow Corning silicone oil with a viscosity ${\nu }_{o}=100\,{\rm{cSt}}$, a surface tension ${\gamma }_{o}=20.9\,{\rm{mN}}/{\rm{m}}$ and a density ${\rho }_{o}=960\,{\rm{kg}}/{{\rm{m}}}^{{\rm{3}}}$. We choose oil to ensure the total wetting of the fibers. To generalize the results obtained for silicone oil, we also use soapy water, a mixture of SDS in water 0.01M (${\nu }_{s}=1\,{\rm{cSt}}$, ${\gamma }_{s}\mathrm{=36.3}\,{\rm{mN}}/{\rm{m}}$, ${\rho }_{s}=1000\,{\rm{kg}}/{{\rm{m}}}^{{\rm{3}}}$). Both liquids totally wet the fibers. This insures that the droplets totally wrap the fiber and that they all adopt a barrel shape under the fiber at each experiment. We use a micropipette to dispense volumes from 0.5 *μ*l to 10 *μ*1 on the fibers.

We use nylon fibers with diameters of 200 *μ*m and 300 *μ*m. For each experiment, two fibers are stretched and attached on a rigid frame at four fixing points that are in the same plane to insure the contact between the fibers. So, the fibers simply touch each other. The fixations guarantee that the fibers cross each other with an angle $\theta$, set at 90° for the major part of the paper and changed at the end to show the influence of this parameter. We fix the frame on a platform and we check that one fiber is horizontal thanks to a laser level. The whole frame is able to rotate on the platform giving a well-defined angle *α* to the initially horizontal fiber.

## Results and Discussion

**Figure 2:**
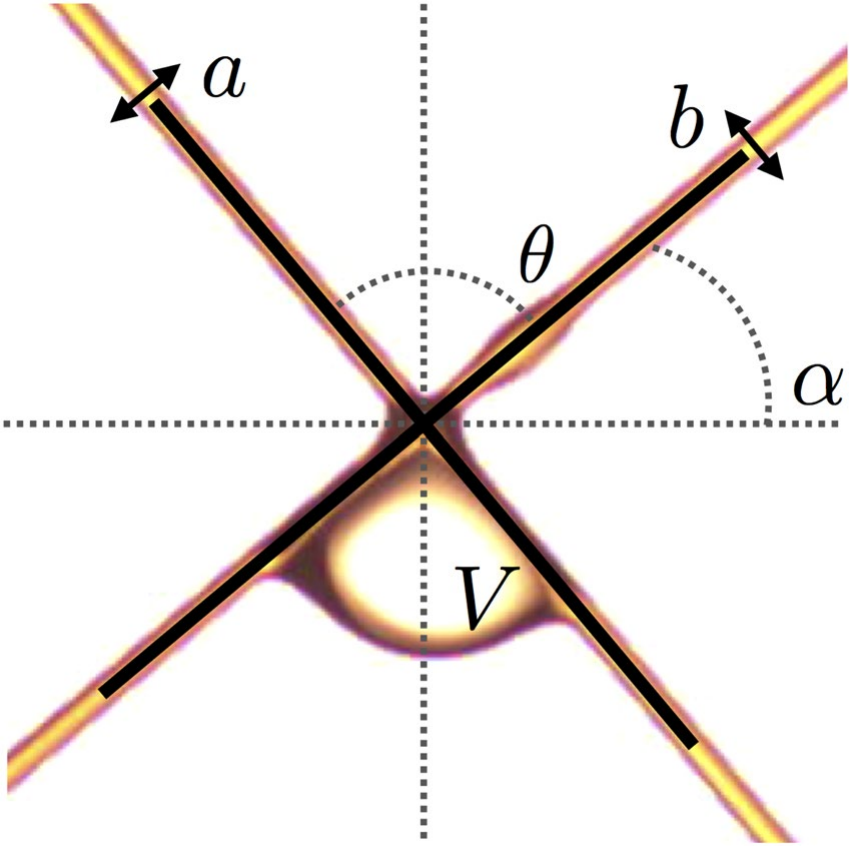
Picture of a 4 *μ*1 oil droplet at the crossing of two fibers of 300 *μ*m in diameter. All necessary parameters are defined: *V* is the volume of the droplet, *α* is the angle between the horizontal and the fiber along which the oil droplet reaches the crossing, $\theta$ is the angle between the fibers, *a* is the diameter of the fiber going from top left to bottom right and *b* is the diameter of the fiber going from top right to bottom left.

Our purpose is to find out how we can control the motion of a droplet on a fiber mesh. We investigate a case which appears simple at first sight: an oil droplet sliding on an inclined fiber and reaching a crossing. In our experiments, we start with nodes made of two perpendicular fibers to study the phenomenon in that specific geometry before generalizing to other angles. The fiber coming from the left and going down to the right has a diameter *a*, the fiber coming from the right has a diameter *b* as shown in Fig. 2. These two fibers cross each other with an angle $\theta$ which is fixed at 90°. The crossing is tilted to demonstrate the importance of the fiber slope. So, we also define the angle *α* as the angle between the right fiber and the horizontal direction. This angle varies from 0° to 90° in steps of 5°. For each angle, a droplet is launched from the right fiber and slides to the crossing where it remains trapped. We choose to deposit and let the droplet slide on the fiber rather than directly placing it on the node in order to mimic as closely as possible the motion of a droplet on a fiber array. Note, the droplet loses some matter while going down due to the fiber coating. However, as we place the droplet close to the node (about 1 cm), the mass loss is negligible. The volume of the initial droplet, *V*, is gradually increased until it detaches. This transition between the trapping and the releasing of the drop defines a critical volume *V*_*max*_.

**Figure 3:**
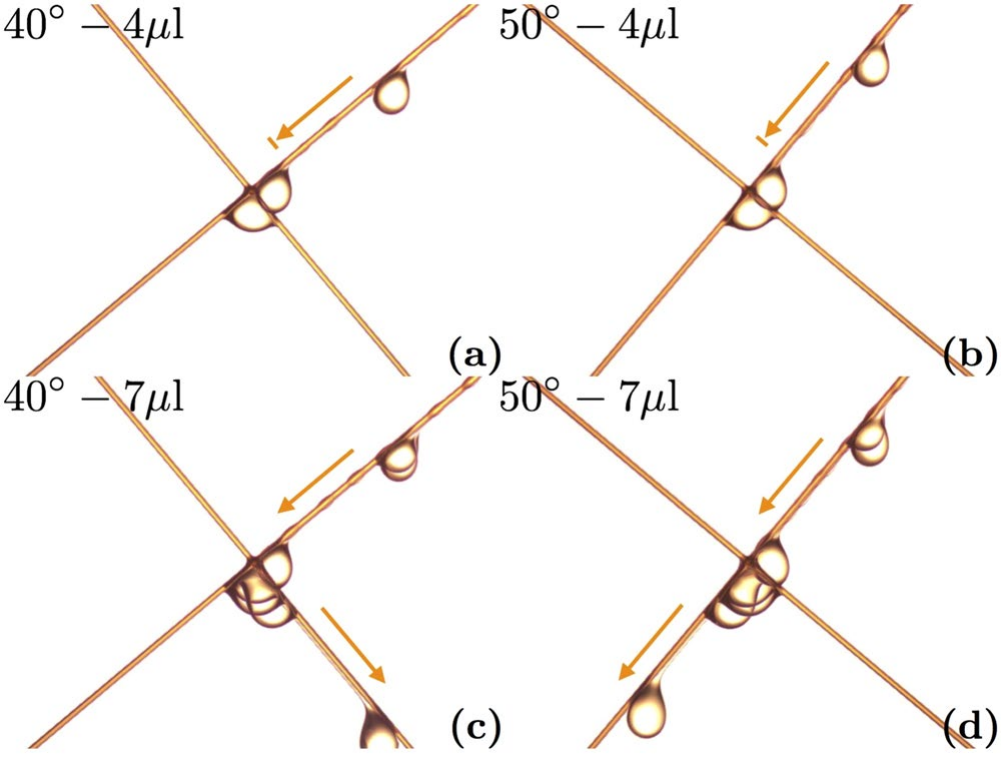
Superimposed pictures of a droplet reaching a crossing made of two 300 *μ*m fibers. Four different situations are shown. In (**a**) and (**c**), the *b* fiber is titled by an angle *α* = 40°. (**a**) The droplet volume is too small to cross the node and the droplet is trapped. Whereas, on (**c**), the droplet is large enough to go through the node and changes fiber. In (**b**) and (**d**), the *b* fiber has a slope *α* = 50°. In (**b**), the droplet is blocked but in (**d**), since the volume is higher, it crosses the node and stays on the same fiber.

First, we look at symmetric nodes (*a* = *b*). For a specific angle *α*, small droplets slide along the right fiber and stop at the crossing as it can be seen from Fig. 3(a),(b). But when droplets become larger, they detach from the node as shown on the picture in Fig. 3(c). It is worth noticing that, for angles smaller than 45°, the droplets always change from the initial fiber to the other one. Conversely, for angles larger than 45°, the droplets with a volume greater than *V*_*max*_ always cross the fiber *a* and stay on the initial fiber. This case is represented in Fig. 3(d). This observation implies that droplets choose the steepest fiber after the crossing. In the case of *α* = 45°, both fibers are exactly equivalent: no fiber is steeper than the other. The droplet randomly chooses one fiber or the other after the crossing.

**Figure 4:**
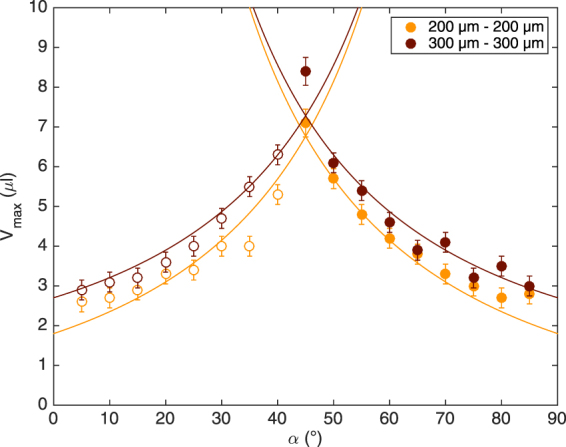
Plot of the maximal volume, *V*_*max*_, that can be held by a symmetric crossing made of 200 *μ*m fibers (in yellow) and of 300 *μ*m fibers (in red). The values are higher for the thicker fibers. For both types of nodes, the volume reaches a maximum for *α* = 45°. The curves correspond to the model we propose based on the force balance. The open circles correspond to droplets changing fiber after the crossing and the disks correspond to droplets staying on the initial fiber.

We determined the maximal volumes, *V*_*max*_, for *α* going from 0° to 90° and for perpendicular fibers of 200 *μ*m and 300 *μ*m in diameter. All our data are gathered in Fig. 4. It can be seen that the maximal volume increases with the angle until it reaches a maximum at 45°. After this optimum, the volume decreases. For angles smaller than 45°, every droplet changes fiber (represented by circles) whereas, for angles larger than 45°, every droplet stays on the initial fiber (represented by disks). The values of *V*_*max*_ obtained for angles smaller and higher than 45° look symmetrical with respect to the maximum. This means that, no matter if the droplet is launched on one fiber (with a slope *α*) or on its complementary fiber (with a slope 90° − *α*), the critical volumes are equal and the final fiber is the same. Moreover, our data indicate that the fiber diameter influences the transition volume: the thicker the fiber, the larger the volume.

**Figure 5:**
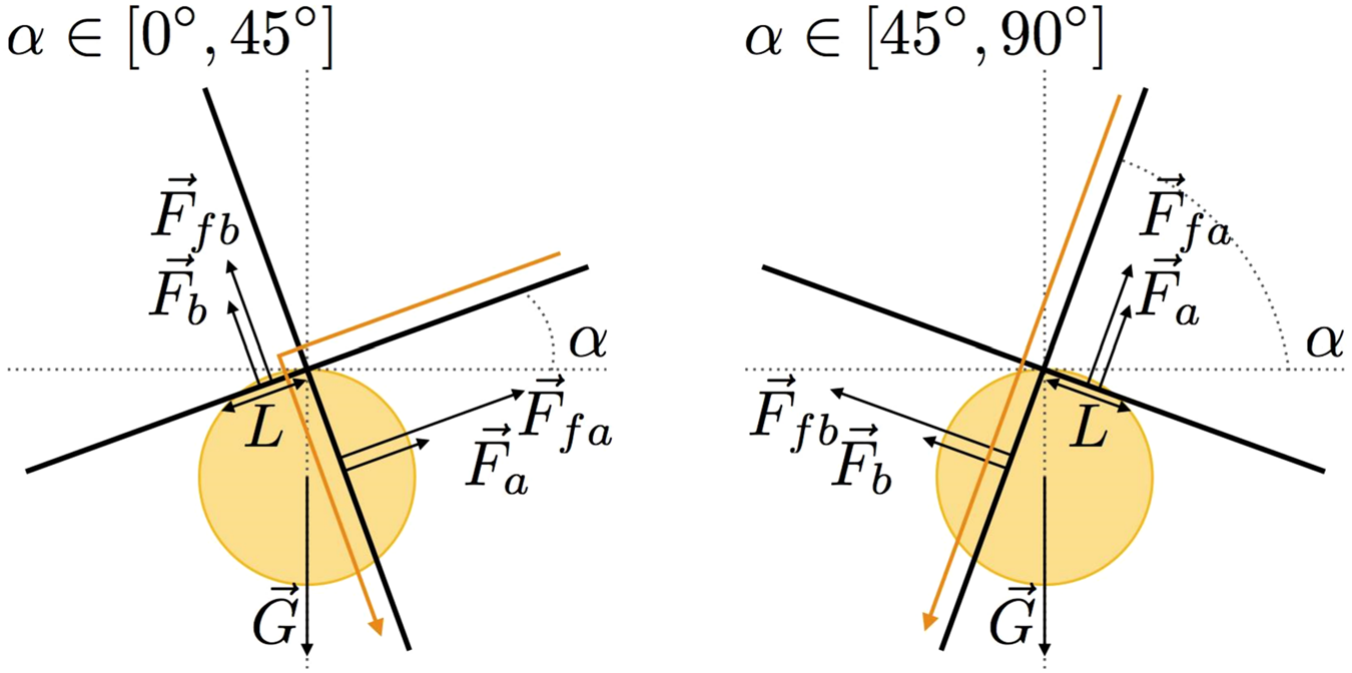
Diagrams of the forces acting on the oil droplets when they leave the crossing. *G* is the weight of the droplet, *F*_*a*_ and *F*_*b*_ are the capillary forces applying along the contact lines and *F*_*fa*_ and *F*_*fb*_ are the forces due to the oil film. *L* is the wetting length of the droplet along the fiber perpendicular to the detachment direction. The orange arrow represents the detachment direction of the drop.

Based on our observations, we propose a model to predict the volume at which a droplet crosses the node. The detachment process is a really slow process. The droplet is drastically slowed down at the crossing. The typical speed of the droplets is about 10^−2^ m/s for the steepest fibers and the largest droplets. The capillary number compares viscosity and capillarity and is defined as ${\rm{Ca}}={\rho }_{o}{\nu }_{o}{\rm{v}}/{\gamma }_{o}$ where v is the speed of the drop. It is found to be equal to $4.6\times {10}^{-2}$ meaning that capillarity overcomes viscosity. Moreover, the Weber number, ${\rm{We}}={\rho }_{o}l{{\rm{v}}}^{2}/{\gamma }_{o}$, is the balance between inertia and capillarity. 1 is the characteristic length of the system that we consider to be the droplet radius (1 = 0.001 m). In our system, one has ${\rm{We}}\simeq 4.6\times {10}^{-3}$. We can therefore conclude that the capillary effects prevail during the whole process. So, we propose a quasi-static model avoiding hydrodynamical forces. In our case, the driving force is the gravity but we can imagine to change or to add other driving forces. What really matters is that these forces overcome a resistive force due to the node as discussed here below. We assume that the droplet has a spherical shape as depicted in Fig. 5. We identify three types of forces acting on the droplet: the weight *G*, the capillary forces *F*_*a*_ and *F*_*b*_ acting on the contact lines on both fibers and the forces due to the presence of a liquid film along the fibers *F*_*fa*_ and *F*_*fb*_. Indeed, prior to the detachment, the droplet surrounds both fibers but when it leaves the node, it has to detach from one of the fibers. There is a resistive force due to the detachment of the droplet from the fiber that is acting against the motion of the droplet. This force is larger both when the surface tension is higher and when the film is wider. Therefore, *F*_*fa*_ and *F*_*fb*_ are directly proportional to the surface tension ${\gamma }_{o}$ and the wetting length *L*. These forces can be seen as forces resisting the droplet detachment.

We consider the balance of all the forces along the detachment direction. We have to distinguish two cases: $\alpha \in [{0}^{\circ },\,{45}^{\circ }]$, when the droplet changes fiber and $\alpha \in [{45}^{\circ },\,{90}^{\circ }]$, when the droplet stays on the fiber.

(i) For $\alpha \in [{0}^{\circ },\,{45}^{\circ }]$, we have

1 $$G\,\cos \,\alpha ={F}_{b}+{F}_{fb}\mathrm{.}$$

The weight can be rewritten as ${\rho }_{o}gV$, the capillary force as $2\pi {\gamma }_{o}b$ as proposed by Lorenceau *et al*.^13^ and the force *F*_*fb*_ as ${\gamma }_{o}L$ where *L* is the wetting length along fiber *b*. As we assume a nearly spherical shape, $L=2{\zeta }^{L}r\,\sin \,\alpha$ where *r* is the droplet radius and ${\zeta }^{L}$ is a fitting parameter that takes into account the deviation of the droplet shape from a spherical shape. Therefore, Eq. (1) can be rewritten as

2 $$\frac{4\pi }{3}{\rho }_{o}g{r}^{3}\,\cos \,\alpha -2{\zeta }^{L}{\gamma }_{o}r\,\sin \,\alpha -2\pi {\gamma }_{o}b=0.$$

There is only a single real solution for this third-degree equation which leads to

3 $$\begin{array}{ccc}r & = & 3\sqrt{\frac{3{\lambda }_{o}^{2}b}{4\,\cos \,\alpha }+\sqrt{{(\frac{3{\lambda }_{o}^{2}b}{4\cos \alpha })}^{2}-{(\frac{{\zeta }^{L}{\lambda }_{o}^{2}\sin \alpha }{2\pi \cos \alpha })}^{3}}}\\  &  & +3\sqrt{\frac{3{\lambda }_{o}^{2}b}{4\,\cos \,\alpha }-\sqrt{{(\frac{3{\lambda }_{o}^{2}b}{4\cos \alpha })}^{2}-{(\frac{{\zeta }^{L}{\lambda }_{o}^{2}\sin \alpha }{2\pi \cos \alpha })}^{3}}},\end{array}$$

where ${\lambda }_{o}=\sqrt{{\gamma }_{o}/{\rho }_{o}g}$ is the capillary length. (ii) For $\alpha \in [{45}^{\circ },\,{90}^{\circ }]$, the projection of the forces on the complementary fiber gives

4 $$G\,\sin \,\alpha ={F}_{a}+{F}_{fa}\mathrm{.}$$

In this case, *L* is the wetting length along the fiber *a* and $L=2{\zeta }^{L}r\,\cos \,\alpha$, which means that

5 $$\frac{4\pi }{3}{\rho }_{o}g{r}^{3}\,\sin \,\alpha -2{\zeta }^{L}{\gamma }_{o}r\,\cos \,\alpha -2\pi {\gamma }_{o}a=0.$$

We obtain a similar equation for the radius of the droplet

6 $$\begin{array}{ccc}r & = & 3\sqrt{\frac{3{\lambda }_{o}^{2}a}{4\,\sin \,\alpha }+\sqrt{{(\frac{3{\lambda }_{o}^{2}a}{4\sin \alpha })}^{2}-{(\frac{{\zeta }^{L}{\lambda }_{o}^{2}\cos \alpha }{2\pi \sin \alpha })}^{3}}}\\  &  & +3\sqrt{\frac{3{\lambda }_{o}^{2}a}{4\,\sin \,\alpha }-\sqrt{{(\frac{3{\lambda }_{o}^{2}a}{4\sin \alpha })}^{2}-{(\frac{{\zeta }^{L}{\lambda }_{o}^{2}\cos \alpha }{2\pi \sin \alpha })}^{3}}}\mathrm{.}\end{array}$$

Based on Eqs (3) and (6), we can calculate the theoretical critical volume ${V}_{max}=\frac{4\pi }{3}{\zeta }^{V}{r}^{3}$ as a function of *α*. ${\zeta }^{V}$ is a geometric pre-factor correcting the theoretical expression of the maximal volume. We consider ${\zeta }^{V}$ as a free parameter for the case, described here above, of two perpendicular fibers with identical diameters. However, once the value of ${\zeta }^{V}$ is obtained, it is kept constant for the rest of the paper. For each node, we fit both branches of the model and we calculate the average fitting parameters. In Fig. 4, the different fits are plotted with ${\zeta }_{200\mu m-200\mu m}^{L}=1.08\pm 0.16$ for nodes made of two fibers of 200 *μm* and ${\zeta }_{300\mu m-300\mu m}^{L}=0.87\pm 0.11$ for fibers of 300 *μm*. We find that ${\zeta }^{V}$ is almost the same for both sets of data and is therefore fixed at ${\zeta }^{V}=0.65\pm 0.12$. Theses parameters are needed to take into account any deviation from the ideal spherical shape of the droplet. A careful observation of the pictures shows that, at the crossing, the droplet is almost a sphere except that the fibers deform and stretch the upper part of the droplet. Assuming a spherical shape can induce an error on both the wetting length (taken into account by ${\zeta }^{L}$) and the volume (taken into account by ${\zeta }^{V}$). The parameter ${\zeta }^{L}$ corrects the theoretical wetting length obtained by assuming that the droplet is a sphere. This parameter may change depending on the fiber diameter, the nature of the liquid or the angle between the fibers. Conversely, the value of ${\zeta }^{V}$ is a constant. By considering the maximal volume as the volume of a sphere of radius *r*, our model overestimates the critical volume and ${\zeta }^{V}$ corrects this bias. Therefore, the value of ${\zeta }^{V}$ is fixed for the rest of the paper and can be seen as a geometric pre-factor.

**Figure 6:**
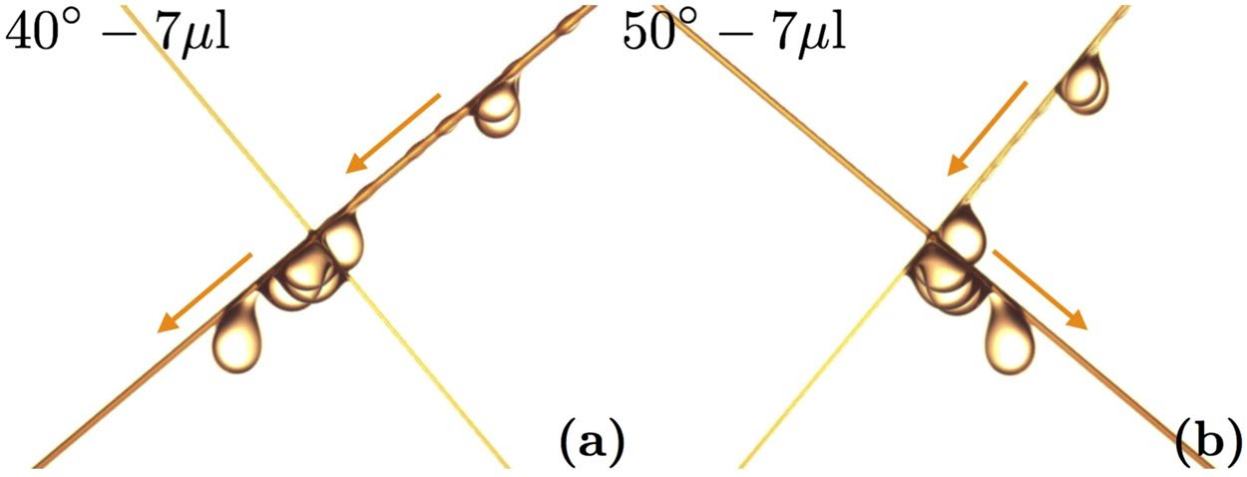
Superimposed pictures of oil droplets crossing asymmetric nodes. On the left, the 300 *μ*m fiber goes from the top right to the bottom left. A droplet goes through a node and surprisingly stays on the initial fiber even though it is not the steepest. On the right, the 300 *μ*m fiber goes from the top left to the bottom right. An oil drop changes from the steepest fiber to the thickest fiber after the crossing. On both pictures, the second fiber has a diameter of 200 *μ*m.

After the study of symmetric nodes, we focus on asymmetric crossings ($a\ne b$). We create intersections of a 200 *μ*m fiber and a 300 *μ*m fiber and we rotate them. We test two different situations: launching the droplet on the thin fiber (named 200 *μ*m–300 *μ*m) or launching it on the thick fiber (named 300 *μ*m-200 *μ*m). We are able to detect the transition as done before. During our experiments on asymmetric nodes, we notice unexpected behaviors. Indeed, unlike the symmetric case where the droplets select the steepest fiber after the crossing, some droplets do not choose the steepest but the thickest fiber. As shown in Fig. 6(a), a droplet glides along a 40° inclined fiber and, instead of changing fiber, it stays on this thick fiber. Another example, in Fig. 6(b), shows that for a 200 *μ*m fiber with a 50° slope, the droplet switches to the 300 *μ*m fiber even though its slope is lower. This means that by choosing the right fiber diameters, we can force the droplet to select the thickest fiber even if it is not the steepest.

**Figure 7:**
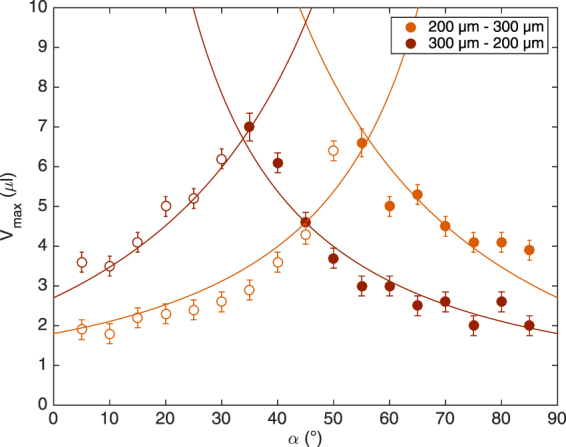
Plot of the maximal volume of the droplet at an asymmetric node made of two different fibers: one of 200 *μ*m in diameter and the other of 300 *μ*m. Data in pale orange correspond to the case 200 *μ*m–300 *μ*m meaning that the droplet is launched on the thin fiber and crosses the thick fiber. Data in dark orange represent the other case 300 *μ*m–200 *μ*m meaning that the drop is initially placed on the 300 *μ*m fiber. The circles correspond to droplets changing fiber after the crossing and the disks correspond to droplets staying on the initial fiber. The curves come from the model developed for symmetric nodes but applied on asymmetric cases.

This phenomenon is obvious when looking at the graph in Fig. 7. The curves have the same general shape as the ones in Fig. 4 but the maxima are shifted. The angle above which the droplets stay on the fiber instead of changing it is no longer $\alpha ={45}^{\circ }$. For the case 300 *μ*m-200 *μ*m, the transition angle is lower meaning that the droplet remains on the thick initial fiber even if the thin fiber is steeper. In the case 200 *μ*m-300 *μ*m, the transition angle is higher. Thus, the droplet switches from the initial fiber to a less steep but thicker fiber. These results show that for a range of angles about $\alpha \in [{30}^{\circ },\,{60}^{\circ }]$, the droplet always chooses the thickest fiber. Out of this range, the droplet selects the steepest fiber.

Moreover, it should be noted that the curves corresponding to 200 *μ*m-300 *μ*m and 300 *μμ*m-200 *μ*m are symmetrical to one another in relation to $\alpha ={45}^{\circ }$. So, for complementary cases (a 300 *μ*m fiber at *α* and a 200 *μ*m fiber at ${90}^{\circ }-\alpha$), the critical volumes are the same and, after the crossing, the behavior of the droplet is also the same. This means that the detachment process is dictated by the configuration of the node rather than by the initial fiber.

We apply the exact same model as the one presented here above. We just specify the suitable fiber diameters *a* and *b*. The curves are exactly the same as the ones is Fig. 4, the only difference is the values of the parameter ${\zeta }^{L}$. As our model does not take into account the influence of the fiber parallel to the detachment direction, we can assume that the values of the parameter for asymmetric nodes are different from the ones obtained with symmetric crossings. We first fit the data that correspond to a droplet crossing a 200 *μ*m fiber (the left branch of the case 200 *μ*m-300 *μ*m and the right branch of the case 300 *μ*m-200 *μ*m) as the force due to the film depends on the wetting length along this fiber. Then, we consider the data for droplets crossing a 300 *μ*m fiber (the left branch of the case 300 *μ*m-200 *μ*m and the right branch of the case 200 *μ*m-300 *μ*m). We obtain ${\zeta }_{200\mu m}^{L}=0.60\pm 0.12$ and ${\zeta }_{300\mu m}^{L}=1.33\pm 0.24$. Note, there is only one fitting parameter as the value of ${\zeta }^{V}$ has been fixed at 0.65 as discussed for the symmetric nodes. We plot the four different branches and we associate them to their corresponding cases as it is shown in Fig. 7. These curves are in good agreement with our data. The model also predicts a maximum volume for angles *α* different from 45°. It confirms that when the node is made of two different fibers, the transition between droplets changing or staying on the initial fiber is no longer at 45°. There is a range of angles in which the relevant parameter is no more the slope of the fiber but its thickness.

After the detailed study of oil droplets sliding on perpendicular fibers, we try to generalize our results for other liquids and other angles between the fibers.

**Figure 8:**
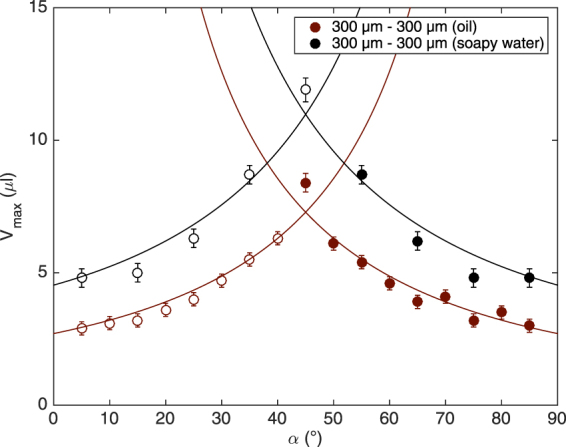
Plot of the maximal volume, *V*_*max*_, that can be held by a symmetric crossing made of 300 *μ*m fibers for oil (in red) and soapy water (in black). The maximal volumes are larger for soapy water than for oil as the surface tension is higher. However, the data have a similar shape and the volumes reach a maximum for $\alpha ={45}^{\circ }$ for both liquids. The circles correspond to droplets changing fiber after the crossing and the disks correspond to droplets staying on the initial fiber. The curves correspond to the model developed for oil and applied for water.

We perform the experiment with soapy water on perpendicular fibers to show the potential influence of a change in surface tension and viscosity. The behavior of these droplets are similar to the one of oil droplets: the droplets always select the steepest fiber after the crossing. The viscosity does not seem to affect the phenomenon. However, the surface tension changes the critical volumes above which the droplet detaches from the node. As the surface tension is higher for soapy water than for oil, the maximum volumes are larger. Figure 8 shows the maximum volume of soapy water in black compared to oil in red for a node made of two perpendicular fibers of 300 *μ*m in diameter. The shape of the graphs is similar and we can apply the model given by Eqs (2) and (5). We only have to consider the parameters of soapy water (${\nu }_{s}$, ${\gamma }_{s}$ and ${\rho }_{s}$) and define the corresponding capillary length ${\lambda }_{s}$. The black curves correspond to the model applied on the soapy water data, they seem to be in good agreement with them. The fitting parameter is ${\zeta }_{s}^{L}=0.60\pm 0.21$ and is comparable to one obtained for oil (${\zeta }_{300\mu m-300\mu m}^{L}=0.87\pm 0.11$). So, the viscosity does not influence the phenomenon whereas the surface tension modifies the maximal volume. The model is able to take the influence of surface tension into account.

**Figure 9:**
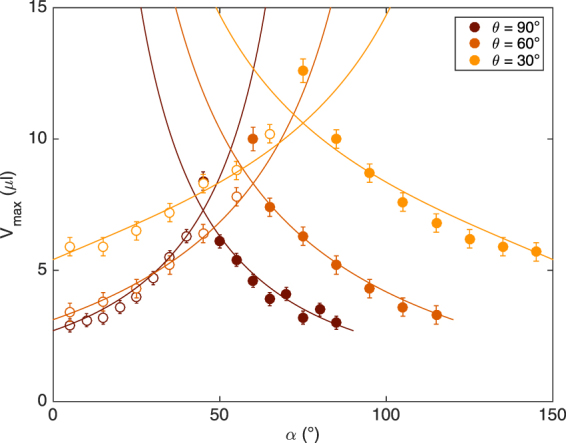
Plot of the maximal volume, *V*_*max*_, that can be held by a symmetric crossing made of 300 *μ*m fibers as a function of the inclination, *α*, of the initial fiber. Data for different angles, $\theta$, between the fibers are presented. The circles correspond to droplets changing fiber after the crossing and the disks correspond to droplets staying on the initial fiber. The droplet behavior is similar for $\theta ={90}^{\circ }{\mathrm{,60}}^{\circ }$ or 30°: for small *α*, the droplet changes fiber, then, when both fibers have the same slope, the droplet chooses one fiber or the other, finally for large *α*, the droplet stays on the initial fiber. The maximum value of *V*_*max*_ is reached when both fibers have the same slope which occurs at different angles *α*. For $\theta ={90}^{\circ },\,{60}^{\circ }$ and 30°, the fibers have the same inclination for $\alpha ={45}^{\circ },\,{60}^{\circ }$ and 75° respectively. The curves correspond to the generalized model given by Eqs (7) and (8).

Finally, we change the angle $\theta$ between the fibers. We use oil droplets on two identical fibers of 300 *μ*m in diameter and we cross them with an angle of 60° and 30°. Then, we rotate the node as done for the previous experiments. Our conclusions for $\theta ={90}^{\circ }$ still hold for other crossing angles. The droplet selects the fiber with the highest slope after the crossing. Moreover, the critical volume increases with the angle *α* until it reaches a maximum and then decreases. The maximum occurs when both fibers have the same slope. For $\theta ={90}^{\circ }$, it corresponds to $\alpha ={45}^{\circ }$ but for $\theta ={60}^{\circ }$ and 30°, this happens when $\alpha ={60}^{\circ }$ and 75°, respectively. As the angle $\alpha$ for which both fibers have the same slope depends on the angle $\theta$ between the fibers, the maximal volume, *V*_*max*_, has to be studied over different ranges of *α*, i.e. from $\alpha ={0}^{\circ }$ to $\alpha ={90}^{\circ }$ for $\theta ={90}^{\circ }$, to $\alpha ={120}^{\circ }$ for $\theta ={60}^{\circ }$ and to $\alpha ={150}^{\circ }$ for $\theta ={30}^{\circ }$. This can be seen from the Fig. 9. The model proposed for perpendicular fibers can be smartly generalized for any angle $\theta$ between the fibers.

(i) For $\alpha \in [{0}^{\circ },\frac{{180}^{\circ }-\theta }{2}]$, we have

7 $$\frac{4\pi }{3}{\rho }_{o}g{r}^{3}\,\sin (\theta +\alpha )-2{\zeta }^{L}{\gamma }_{o}r\,\sin \,\alpha -2\pi {\gamma }_{o}b=0.$$

(ii) And for $\alpha \in [\frac{{180}^{\circ }-\theta }{2}{\mathrm{,180}}^{\circ }-\theta ]$, we have

8 $$\frac{4\pi }{3}{\rho }_{o}g{r}^{3}\,\sin \,\alpha -2{\zeta }^{L}{\gamma }_{o}r\,\sin (\theta +\alpha )-2\pi {\gamma }_{o}a=0.$$

These equations lead to generalized expressions of the radius $r$ and the volume ${V}_{max}=\frac{4\pi }{3}{\zeta }^{V}{r}^{3}$. This more general version of the model is applied to our data and the corresponding curves are plotted on Fig. 9. The fitting parameters are ${\zeta }_{{60}^{\circ }}^{L}=1.25\pm 0.06$ and ${\zeta }_{{30}^{\circ }}^{L}=1.73\pm 0.10$ (${\zeta }^{V}$ is still fixed at 0.65). The model seems to be in good agreement with our data for $\theta ={60}^{\circ }$ and 30°. Regarding the symmetry of the problem, our conclusions can be extended to the cases of $\theta ={120}^{\circ }$ and 150°. The values of ${\zeta }^{L}$ increase when the angle $\theta$ decreases, starting from 0.87 for 90° up to 1.73 for 30°. This can easily be understood. When the angle between the fibers decreases, the droplet gradually loses its sphericity and spreads along the fiber that is perpendicular to the detachment direction leading to an increase of the wetting length and consequently to an increase of ${\zeta }^{L}$. Our model seems to be robust regarding both the nature of the liquid and the angle between the fibers.

The results allow us to understand how a droplet progresses on a fiber mesh. For an array made of perpendicular fibers, the droplet detaches along the steepest fiber. This means that if the droplet is initially on the most inclined fiber, it remains on it during its motion, crossing after crossing. If not, at the first crossing, it changes to the steepest fiber and stays on it afterwards. So, by rotating the whole array, we can switch from one way to the other. Another possibility, which may be even more convenient, is to pick the right fiber diameters. Indeed, from an appropriate angle, the droplet always changes from one fiber to a thicker one. In Fig. 1, we created an array made of six different fibers. This network is rotated through 45°. Three fibers go from the top left to the bottom right with increasing diameters (160 *μ*m, 250 *μ*m and 350 *μ*m) and three other fibers go from the top right to the bottom left with increasing diameters (200 *μ*m, 300 *μ*m and 450 *μμ*m). The droplet is placed on the thinnest fiber. As all fibers have the same slope, the droplet always chooses the thickest fiber. Given the way we built the network, the droplet switches from one fiber to the other at each node. As shown in Fig. 1, the droplet literally zigzags on the fiber array.

## Conclusion

In this paper, we proposed one of the first studies focusing on the droplet motion on a fiber network. We showed that both the fiber steepness and the fiber diameter are crucial parameters when talking about fiber-based microfluidics. We focused on the crossings of fiber arrays and we determined the critical volume above which droplets go through the nodes. We proposed a model based on the competition between the gravity and the forces due to the film that wets both fibers with a single fitting parameter. This model is able to predict the detachment volume. Moreover, it could be advantageously implemented for situations involving other driving forces. On a network made of perpendicular identical fibers, we revealed that droplets always opt for the steepest fiber at each crossing. When fibers have different diameters, we highlighted that, for certain angles, droplets select the thickest fiber regardless of its slope. We showed that our model still works for other liquids and other angles between the fibers. The only limitation is the use of liquids that totally wet the fibers. Further experiments should be performed to extend our findings to droplets that only partially wet the fibers. In that case, wetting properties of the substrate are crucial (its chemistry, its roughness,…) as well as the droplets themselves. The way the droplet is placed on the fiber influences the droplet behavior due to the wetting hysteresis. The contact angle difference between the top and the bottom of the droplet may prevent the droplet to slide. This problem requires a specific attention. However, our results prove that by cleverly designing a fiber network, a droplet can easily be guided through an asymmetric fiber maze.

This convenient technique is an opportunity to develop an easy fiber-based microfluidics which does not require pumps, microchannels or expensive equipments. Moreover, this is a chance to optimize the fog harvesting simply by changing the geometry of fog nets which are currently developed.

## References

[1] Pan Z (2016). The upside-down water collection system of Syntrichia caninervis. Nature plants.

[2] Ju J (2012). A multi-structural and multi-functional integrated fog collection system in cactus. Nature commun..

[3] Zheng Y (2010). Directional water collection on wetted spider silk. Nature.

[4] Hou Y, Chen Y, Xue Y, Zheng Y, Jiang L (2012). Water Collection Behavior and Hanging Ability of Bioinspired Fiber. Langmuir.

[5] Wang Y, Zhang L, Wu J, Hedhilib MN, Wang P (2015). A facile strategy for the fabrication of a bioinspired hydrophilic: superhydrophobic patterned surface for highly efficient fog-harvesting. J. Mater. Chem. A.

[6] Chen Y, Wang L, Xue Y, Jiang L, Zheng Y (2007). Bioinspired tilt-angle fabricated structure gradient fibers: micro-drops fast transport in a long-distance. EPL.

[7] Chen Y, Li D, Wang T, Zheng Y (2016). Orientation-Induced Effects of Water Harvesting on Humps-on-Strings of Bioinspired Fibers. Scientific Reports.

[8] Piroird K, Clanet C, Lorenceau E, Quéré D (2009). Drops impacting inclined fibers. Journal of Colloid and Interface Science.

[9] Kim, H. *et al*. Water harvesting from air with metal-organic frameworks powered by natural sunlight, *Science* (2017). 10.1126/science.aam8743 28408720

[10] Bai H (2011). Large-Scale Fabrication of Bioinspired Fibers for Directional Water Collection. small.

[11] Park K-C, Chhatre SS, Srinivasan S, Cohen RE, McKinley GH (2013). Optimal Design of Permeable Fiber Network Structures for Fog Harvesting. Langmuir.

[12] Seo D, Lee J, Lee C, Nam Y (2016). The effects of surface wettability on the fog and dew moisture harvesting performance on tubular surfaces. Scientific reports.

[13] Lorenceau E, Clanet C, Quéré D (2004). Capturing drops with a thin fiber. Journal of Colloid and Interface Science.

[14] Lorenceau, E. Senden, T. & Quéré, D. Wetting of fibers, *Molecular Gels. Materials with Self-Assembled Fibrillar**Networks*, 223–237 (2006).

[15] Mullins BJ, Agranovski IE, Braddock RD, Ho CM (2004). Effect of fiber orientation on fiber wetting processes. Journal of Colloid and Interface Science.

[16] Huang Z, Liao X, Kang Y, Yin G, Yao Y (2009). Equilibrium of drops on inclined fibers. Journal of Colloid and Interface Science.

[17] Gilet T, Terwagne D, Vandewalle N (2010). Droplets sliding on fibres. Eur. Phys. J. E.

[18] Gilet T, Terwagne D, Vandewalle N (2009). Digital microfluidics on a wire. Applied physics letters.

[19] Weyer F, Lismont M, Dreesen L, Vandewalle N (2015). Compound droplet manipulations on fiber arrays. Soft Matter.

[20] Sauret A, Bick AD, Duprat C, Stone HA (2014). Wetting of crossed fibers: multiple steady states and symmetry breaking. EPL.

[21] Sauret A, Boulogne F, Soh B, Dressaire E, Stone HA (2015). Wetting morphologies on randomly oriented fibers. Eur. Phys. J. E.

[22] Py C, Bastien R, Bico J, Roman B, Boudaoud A (2007). 3D aggregation of wet fibers. EPL.

[23] Duprat C, Protière S, Beebe AY, Stone HA (2012). Wetting of flexible fibre arrays. Nature.

